# Play-solicitation gestures in chimpanzees in the wild: flexible adjustment to social circumstances and individual matrices

**DOI:** 10.1098/rsos.160278

**Published:** 2016-08-24

**Authors:** Marlen Fröhlich, Roman M. Wittig, Simone Pika

**Affiliations:** 1 Humboldt Research Group ‘Evolution of Communication’, Max Planck Institute for Ornithology, Eberhard-Gwinner-Straße 9, 82319 Seewiesen, Germany; 2 Primatology Department, Max Planck Institute for Evolutionary Anthropology, Deutscher Platz 6, 04103 Leipzig, Germany; 3 Taï Chimpanzee Project, Centre Suisse de Recherches Scientifiques, BP 1303 Abidjan 01, Ivory Coast

**Keywords:** gesture, play, flexibility, self-handicapping, great ape, chimpanzee

## Abstract

Social play is a frequent behaviour in great apes and involves sophisticated forms of communicative exchange. While it is well established that great apes test and practise the majority of their gestural signals during play interactions, the influence of demographic factors and kin relationships between the interactants on the form and variability of gestures are relatively little understood. We thus carried out the first systematic study on the exchange of play-soliciting gestures in two chimpanzee (*Pan troglodytes*) communities of different subspecies. We examined the influence of age, sex and kin relationships of the play partners on gestural play solicitations, including object-associated and self-handicapping gestures. Our results demonstrated that the usage of (i) audible and visual gestures increased significantly with infant age, (ii) tactile gestures differed between the sexes, and (iii) audible and visual gestures were higher in interactions with conspecifics than with mothers. Object-associated and self-handicapping gestures were frequently used to initiate play with same-aged and younger play partners, respectively. Our study thus strengthens the view that gestures are mutually constructed communicative means, which are flexibly adjusted to social circumstances and individual matrices of interactants.

## 1. Introduction

Humans' unique creativity and innovation skills have been suggested to be longer-term outcomes and benefits of playfulness [1]. The high impact of play on evolutionary and ontogenetic development has further been emphasized by studies on humans' closest living relatives, the non-human primates (hereafter primates), showing that investments in play can take ontogenetic priority over growth with persisting consequences for life history [2]. Play has been defined as repetitious behaviour out of ‘serious' contexts that does not serve an immediate purpose [3]. It represents an essential building block to the development of physical and social tactics in life, by which immature individuals learn to explore and manipulate their physical and social worlds [4,5]. Play behaviour consists of solitary and social play [6] and often involves the use of objects [7]. Importantly, first object play occurs in human children at the age of 12–18 months, when they also start to combine their first spoken words with gestures [8,9]. Evolutionary explanations for play behaviour focused on its function to provide practice or training for specific behaviours needed as adult individuals, such as for instance food processing or tool use [10]. Although tool use is a relatively rare phenomenon in the primate order [11], one of our closest living cousins, chimpanzees (*Pan troglodytes*), not only regularly engage in tool use and manufacture in a variety of contexts (e.g. feeding, self-maintenance and social contexts) [12,13] but also frequently engage in object play [7]. For instance, chimpanzees of the *Kanyawara* community in Uganda have been observed to engage in stick-carrying behaviour [14], which has been suggested to represent a rudimentary variant of doll play observed in human children. Lonsdorf *et al.* [15] highlighted the role of play in chimpanzee infancy, showing that solitary and social play comprise about one-third of an infant's observation time at particular developmental stages.

Overall, social play interactions offer a unique platform for individuals to gain experience in relation to (i) physical and social characteristics of conspecifics, (ii) behavioural distinctions (e.g. playful versus agonistic behaviours), and (iii) different social constellations and contexts [16–18]. However, social play may serve different functions in individuals of different species and also in individuals of different ages and sex within the same species (see for a review [19]). Furthermore, one of the dominant functions of social play is seen in the learning to use and decode communicative signals [20] via elements that promote the initiation or continuation of play interaction [19]. Play signals in great apes include not only a large variety of communicative means, such as gestures [21,22], vocalizations and facial expressions [5], but also spatial cues and relaxedness of movement [23]. Together, these elements serve as meta-communicatory devices to clarify the meaning of ambiguous, potentially agonistic behaviour, especially as play bouts become more intense [24].

Although a number of great ape studies have focused on the role of facial expressions [23,25] and gestural variety [26–29] used during play interactions, virtually nothing is known about how flexibly these communicative means can be adjusted to individual matrices and social circumstances of the interactants. Flexible signalling, a key hallmark of cognitive complexity in a communicative system, has thus far mainly been demonstrated in great apes by means–end dissociation between the signal and context [21,30,31] and combination of signals into sequences [32–34]. Moreover, there has been a strong bias towards studies on play signalling conducted on captive individuals [23,25,35]. Systematic, quantitative comparisons in natural environments taking into account within-species variability, that is including several communities, are still severely under-represented in research on great ape communication (however, see [36,37]).

Here, we carried out the first study addressing the influence of demographic factors and kin relationships on signalling by examining play interactions in two communities of different subspecies of chimpanzees (*P. troglodytes schweinfurthii* and *P. troglodytes verus)* in the wild (*Kanyawara*, Kibale National Park, Uganda, and *Taï South*, Taï National Park, Côte d'Ivoire). Our overall goal was to examine whether chimpanzees are able to adjust their communication to specific attributes of conspecifics (age, sex, kin relationship). In our study, we investigated gestural signals since they (i) are frequent and variable means to initiate social play [21], and (ii) can be reliably recorded in natural environments despite visibility often being limited [36]. We particularly focused on the single communicative function of play initiation only, since keeping the behavioural outcome (i.e. social play) constant enabled us to investigate gestures with the same meaning [36,38,39]. To allow for generalization of between- and within-individual comparison, behavioural data were collected in two distinct field periods per study site across two consecutive years. We thus implemented a developmental approach, which presents an important methodological tool for understanding the cognitive prerequisites underlying different communicative skills [29,40] but has so far only rarely been employed in natural settings [36,37].

Specifically, we addressed the following three questions: First, do age and sex influence the use of play-soliciting gestures? To answer this question, we investigated whether the age and sex of signallers had an effect on the production of audible, tactile and visual gesturing (for categories, see [21]). Since it has been shown that young chimpanzees undergo a developmental shift from actions and tactile to visual [36,41] and audible gestural communication [40], we expected higher frequencies of audible and visual gestures with increasing age. In addition, studies on primates and some other mammal species revealed pronounced sex differences in play behaviour, showing that males engage in more play-fighting and play more vigorously than females (see for a review [42]). This sex difference also might have a strong effect on the employment of signals, but, if present in wild chimpanzees, needs to be disentangled from sex differences with regard to play intensities.

Second, does a prevailing kin relationship between interactants influence the use of gesture modality (audible versus tactile versus visual gesturing)? To address this question, we distinguished between the following play dyad constellations: infant–mother dyads, infant–maternal kin dyads and infant–non-kin dyads (i.e. comprising partners other than mother and maternal kin). Since chimpanzee societies are characterized by a promiscuous mating system, fission–fusion dynamics and male philopatry [43–46], maternal kin relationships are the only recognized bonds that are stable until emigration [47,48]. Hence, the degree of familiarity differs between the partner constellations in different play dyads, since infants have a higher chance of interacting with their maternal siblings (and in some cases aunts and grandmothers) than with paternal siblings and non-related individuals [49]. We predicted that chimpanzees take these relationships into account by predominantly employing tactile gestures in those dyads with the most stable association patterns (i.e. mother–infant dyads). Gestures that involve body contact (i.e. tactile gestures) and are of relatively high risk might be reserved for interactions with more familiar individuals, such as mothers and maternal kin [41,50]. They qualify as honest, costly signals which are more expensive in fitness terms than need be to convey the necessary information [51–53]. They thus should be employed only when predictable outcomes between interactants have been established in previous frequent interactions and in familiar settings, where tactile gestures can be used in different manners without risk. On the other hand, if chimpanzees tailor their gestural production based on previous experiences and interactions with other individuals, visual and audible signals should mainly be used in infant–non-kin dyads.

Third, do chimpanzees take into consideration recipients' attributes such as age, sex and the kin relationship to the signaller? To address this question, we focused on two specific subsets of play-soliciting gestures, so-called object-associated gestures [54] and self-handicapping [19]. Object-associated gestures are gestures accompanied by mobile and immobile objects (e.g. branch shaking^1^). Self-handicapping gestures include signalling postures (e.g. lying in a supine position) that reduce the signaller's probability of achieving its tactical objective in play [55]. If chimpanzees take into consideration distinct attributes of conspecifics, we expected that gesture-object combinations, which may represent more vigorous and straightforward strategies than self-handicapping, would be directed preferentially at partners of the same relative size (i.e. age). Contrarily, we expected to find a higher frequency of self-handicapping gestures in dyads with a relatively large age difference. However, if object-associated and self-handicapping gestures are merely a means of employing different gesture types and forms, we predicted to find a uniform distribution of these signals across all studied dyads.

## 2. Material and methods

### 2.1. Study sites and subjects

The study was conducted at two different chimpanzee communities in the wild: *Kanyawara* in Kibale National Park, Uganda and *Taï South* in Taï National Park, Côte d'Ivoire. Detailed descriptions of the study areas can be found in Wrangham *et al.* [56] and Boesch & Boesch-Achermann [46], respectively. During the two study periods, the size of the *Kanyawara* group varied between 53 and 56 individuals, and 26 and 33 individuals in *Taï South*, respectively. In addition, we had access to long-term data concerning the chimpanzees' demography and relatedness. We observed play interactions of a total of 16 infants (10 from *Kanyawara*, 6 from *Taï South*) with their mothers and conspecifics, with ages ranging from 9 to 74 months (electronic supplementary material, table S1). Conspecifics that the infants interacted with during social play included 10 juveniles/sub-adults (three females, seven males) and 11 adult individuals (eight females, three males) at *Kanyawara*, and 8 juveniles/sub-adults (one female, seven males) and four adult individuals (four females, no males) at *Taï South.* In terms of age classes, individuals were categorized as juvenile/sub-adults if aged between 6 and 13/15 years (females/males) and adults if aged 14/16 years or older (females/males) [46].

### 2.2. Data collection

Observations were made on chimpanzees during four periods between October 2012 and June 2014 (*Kanyawara*: Mar–May 2013; Mar–June 2014; *Taï South*: Oct–Dec 2012; Oct–Dec 2013). We used a focal next to a behaviour sampling approach, that is one individual was observed for a set period of time, while play behaviour of non-focal individuals in the immediate proximity was also recorded [57]. All play interactions of infants (i.e. mother–infant interactions and infant–conspecific interactions) were recorded using a digital high-definition camera (Canon HF M41) with an external unidirectional microphone (Sennheiser K6). This method resulted in a total of 81.9 hours (*Kanyawara*: 44.3, mean ± s.d. per infant = 6.6 ± 4.6; *Taï South*: 37.6, mean ± s.d. = 10.7 ± 5.4, see also electronic supplementary material, table S1) of video footage of play interactions recorded during approximately 1154 hours (*Kanyawara*: 557, *Taï South*: 597) of focal observations.

### 2.3. Coding procedure

To establish the signal repertoires of chimpanzees used to solicit play and enable subsequent analyses, a total of 643 high-quality video files of play interactions were coded using the program Adobe Premiere Pro CS4 (v. 4.2.1.). Behavioural definitions were based on established ethograms of two long-term studies of Eastern Chimpanzees at *Gombe* [22] and *Mahale* [58] and several gesture studies [21,59,60]. A specific coding scheme was developed based on parameters used in previous work on great ape gesturing [28,36,61]. ‘Play solicitations' comprised dyadic play initiation from the start of a play bout, and also dyadic play reinitiation after social play was paused or interrupted by a third individual for at least 10 s. While coding all play interactions, we differentiated play-soliciting gestures from physical actions. An action was defined as any behaviour that resulted in play through direct manipulation of another's body via physical force (e.g. throw on) or one's own locomotion (e.g. move backwards). Contrarily, a gesture was defined as directed, mechanically ineffective movement of the extremities, the body or body postures that elicited (requested) a voluntary response by the recipient [62]. For our analyses, we only included play-initiating gestures that were accompanied by key characteristics of intentional communication: sensitivity to the recipient's attentional state, response waiting, apparent satisfaction of the signaller and goal persistence (for definitions, see [36,63,64]). Signals were clustered into three categories: audible (signals generate a sound while being performed, e.g. slap ground), tactile (signals include physical contact with the recipient, e.g. touch) and visual (signals generate a mainly graphic component, e.g. raise arm) gestures [28]. To identify play solicitations, the behaviour of both the signaller and the recipient throughout the interaction, from first initiating action/gesture until the start of play, was taken into account to assess the success of communicative attempts [65]. Finally, for each signal or action case, we coded the following parameters: signaller's and recipient's age (range = 0–57 years), if signaller = infant: infant age (range = 9–69 months), age class relative to recipient (three levels: same age class, older age class, younger age class), sex of signaller and recipient (two levels: male, female), kin relationship between play partners (three levels: infant–mother, infant–maternal kin, infant–non-kin); play intensity (three levels: low—touching and tickling, intermediate—wrestling and biting, high—rough and tumble). Fifteen per cent of all coded interactions were coded for accuracy by a second observer and tested using the Cohen's *κ* coefficient to ensure inter-observer reliability [57]. The following results were found: a ‘very good’ level of agreement for play intensity (*κ* = 0.868), gesture type (*κ* = 0.827), signal category (*κ* = 0.884), object-associated gesture (*κ* = 0.916) and self-handicapping gesture (*κ* = 0.886) and a ‘good’ level of agreement for intentional usage of signal (*κ* = 0.703).

### 2.4. Analyses

To assess the influence on sampling size, we plotted the cumulative numbers of observed gesture types over time for each study site. If an asymptote was reached (i.e. no further gesture types were observed), we concluded that we had observed the individual's full repertoire for the communicative function of play solicitation [36].

#### 2.4.1. Model specification

To test to which extent sex, age and the kin relationship between the play partners influenced (1) *intensity* of solicited play, (2) frequency of gestures overall (response variables: *audible, tactile, visual* gesture), (3) frequency of *object-associated* gestures, and (4) frequency of *self-handicapping* gestures, we used generalized linear mixed models (GLMMs; [66]) with a Poisson (1) or binomial error structure (2–4) and log (1) or logit link function (2–4). Since infant age varied considerably between infants, we used the method of within-subject centring [67] to tease apart whether the effect of infant age was particularly relevant within and/or between infants. Hence, we included into the model the average age of each infant (being constant across all data points of the respective individual; between-age) and also the difference between the infant's actual age and its average age (within-age). To rule out that age effects do not simply result from higher rates of conspecific-directed signalling with age, we initially included the interactions between relation and both age variables into the model (analyses 1 to 3, removed if non-significant). To control for confounding effects, we also included site as further fixed effect into the model. As random effects (intercepts) we included the identities (ID) of signaller, recipient and the play dyad. To keep type 1 error rates at the nominal level of 5%, we also included the random slopes components of within-age, age difference, sex of signaller/recipient and kinship within signaller ID and/or recipient ID [68,69]. For the other fixed effects, we did not include random slopes because they were usually constant within signaller and recipient ID. We also did not include correlations between random slopes and random intercepts to keep model complexity at an acceptable level and because neglected random slopes do not compromise type 1 error rates [69].

#### 2.4.2. Model implementation

The models were implemented in R [70] using the function *glmer* of the package lme4 [71]. To test the overall significance of our key test predictors [72], we compared the full models with the respective null models comprising only the control predictor (study site) and all random effects using a likelihood ratio test [73]. Prior to running the models, we *z*-transformed between-age, within-age and age difference [74]. To control for collinearity, we determined variance inflation factors (VIF; [75]) from a model including only the fixed main effects using the function *vif* of the R package car [76]. This revealed collinearity to not be an issue (maximum VIF = 1.49). Confidence intervals were derived using the function *sim* of the R package arm [77]. Tests of the individual fixed effects were derived using likelihood ratio tests (R function *drop1* with argument ‘test’ set to ‘Chisq’). All statistical analyses were performed using the R statistical package, version R.3.1.1 [70], with a level of significance set at 0.05.

#### 2.4.3. Datasets

For each of our four analyses, we used different datasets: for analyses 1 and 2, we included play signals of infants towards all possible play partners, including mothers, and used age and sex of the signaller as well as relation to the recipient (mother, maternal kin, non-kin) as fixed effects (for details see dataset in electronic supplementary material, S2). Analysis 3 used the same dataset, but here we excluded the few gestures towards mothers, since object-associated gestures did not seem to play a role in infant–mother play solicitation. Here, we additionally included age difference and sex of the recipient as additional test predictors. Finally, for analysis 4, we included gesture cases by *or* towards infants to examine the role of self-handicapping (i.e. signallers could be of all age classes, for details see dataset in electronic supplementary material, S3). This analysis used the same test predictors as utilized in analysis 3.

## 3. Results

### 3.1. Dataset of play-solicitation gestures

The coding of the dataset resulted in a total of 1174 gesture cases (*Kanyawara*: *N* = 761; *Taï South*: *N* = 413), of which 109 were audible (*Kanyawara*: *N* = 74; *Taï South*: *N* = 35), 646 tactile (*Kanyawara*: *N* = 417; *Taï South*: *N* = 229) and 419 visual gestures (*Kanyawara*: *N* = 270; *Taï South*: *N* = 149; see Material and methods for detailed gesture definitions and criteria used to infer intentional use). Among the observed gesture cases, we found a total of 229 object-associated gestures (*Kanyawara*: *N* = 125; *Taï South*: *N* = 104), which equals 19.5% of all coded gestures. In addition, we identified 74 cases of self-handicap gestures (*Kanyawara*: *N* = 27; *Taï South*: *N* = 47), which corresponds to 6.3% of all gesture cases. To ensure that repertoires had approached and/or reached asymptote, we plotted the cumulative repertoire of gestures over observation time. The results showed that the cumulative repertoires at *Kanyawara* and *Taï* South reached the asymptote after 14 and 20 days of observation, respectively (electronic supplementary material, figure S1). Hence, we assumed that the approximate repertoire of play-soliciting gestures has been captured at both study sites.

### 3.2. Overview on gesture types in play solicitation

To examine which gestures chimpanzees used to initiate play, we compiled gesture repertoires for different age classes (infants, juveniles/adolescents and adults; for classification see Material and methods) of the two sites separately. Across study sites, we identified a total of 48 gesture types. Out of the 48 gesture types, three were audible, 24 were tactile and 21 were visual (for definitions of gesture types, see electronic supplementary material, table S2). We identified five group-specific gesture types: head butt, hide self, present back, present leg, shake head (*Kanyawara-*unique: *N* = 4, *Taï South-*unique: *N* = 1, see electronic supplementary material, table S3). With regard to age classes, across sites infants produced all 48 identified gestures (*Kanyawara: N* = 47, *Taï South: N* = 49). In the juvenile/sub-adult class the number decreased, with a total of 37 gesture types used across sites (*Kanyawara: N* = 32*, Taï South: N* = 27). Finally, the adult age class, including mothers, showed the smallest repertoire with a total of 25 gesture types across sites (*Kanyawara: N* = 24*, Taï South: N* = 16). A detailed overview on gesture types used in play interactions with regard to age class and community/study site can be found as electronic supplementary material, table S3.

### 3.3. Influence of demographic parameters and kin relationship on intensity of initiated play

Before examining the effect of sex on play-solicitation gestures, we analysed whether male (*N* = 10) and female (*N* = 6) chimpanzee infants differed with regard to the intensities of play solicited (*N* = 1157). Males produced on average 52.5 ± 34.4% (mean ± s.d.) gestures resulting in soft play bouts (low intensity), 28.0 ± 23.5% resulting in wrestle bouts (intermediate intensity) and 19.5 ± 24.5% resulting in rough and tumble bouts (high intensity). Females produced on average 68.1 ± 21.0% gestures resulting in soft play bouts, 30.0 ± 19.7% resulting in wrestle bouts, but only 1.8 ± 3.5% resulting in rough and tumble bouts (see also electronic supplementary material, figure S2). We used GLMMs to test the effects of signaller's sex, age (i.e. both between- and within-age) and a prevailing kin relationship to the recipient (i.e. mother, maternal kin and non-kin) as well as the interactions between kin relationship and age (comprising two interaction terms) on play intensity in infant chimpanzees. Overall, the test predictors had a clear impact (likelihood ratio tests comparing null and the full model (LRT): *χ*^2^ = 21.515, d.f. = 7, *p* = 0.003). After removal of the (non-significant) two interactions, our results showed that intensity of play was significantly higher in older signallers (between-age: estimate ± s.e. = 0.152 ± 0.048, *χ*^2^ = 8.210, d.f. = 1, *p* = 0.004) and significantly lower in interactions with mothers (relation/mother: −0.281 ± 0.089, *χ*^2^ = 9.892, d.f. = 1, *p* = 0.002). There was no effect of signaller's sex (sex/male: 0.004 ± 0.090, *χ*^2^ = 0.002, d.f. = 1, *p* = 0.968), maternal kinship (relation/kin: −0.063 ± 0.104, *χ*^2^ = 0.378, d.f. = 1, *p* = 0.539) or study site (site/*Taï South*: −0.119 ± 0.090, *χ*^2^ = 1.783, d.f. = 1, *p* = 0.182). For further details on model output, see electronic supplementary material, table S4.

### 3.4. Influence of demographic parameters and kin relationship on play solicitation

We used GLMMs to test whether the effects of sex, age (i.e. both between- and within-age) and kin relationship to the recipient (i.e. mother, maternal kin and non-kin) as well as the interactions between kin relationship and age (comprising two interaction terms) affected the production of visual, tactile and audible gestures in infant chimpanzees. Overall, the test predictors had a clear impact in all three models (LRT for audible gesturing: *χ*^2^ = 28.676, d.f. = 7, *p* < 0.001; tactile gesturing: *χ*^2^ = 14.976, d.f. = 7, *p* = 0.036; visual gesturing: *χ*^2^ = 21.906, d.f. = 7, *p* = 0.003).

Concerning audible gesturing, we found a significant interaction between mother-directed signalling and age (relation/mother × within-age: 6.132 ± 4.352, *χ*^2^ = 4.117, d.f. = 1, *p* = 0.042). After removal of the other (non-significant) interaction, our results showed these signals were produced significantly more by older infants (between-age: 0.748 ± 0.209, *χ*^2^ = 11.256, d.f. = 1, *p* = 0.001). There was no effect of signaller's sex (sex/male: −0.010 ± 0.613, *χ*^2^ = 0.002, d.f. = 1, *p* = 0.990), maternal kinship (relation/kin: −0.900 ± 0.529, *χ*^2^ = 2.738, d.f. = 1, *p* = 0.098) or study site (site/*Taï South*: −0.424 ± 0.508, *χ*^2^ = 0.716, d.f. = 1, *p* = 0.398) on audible gesturing.

**Figure 1.**
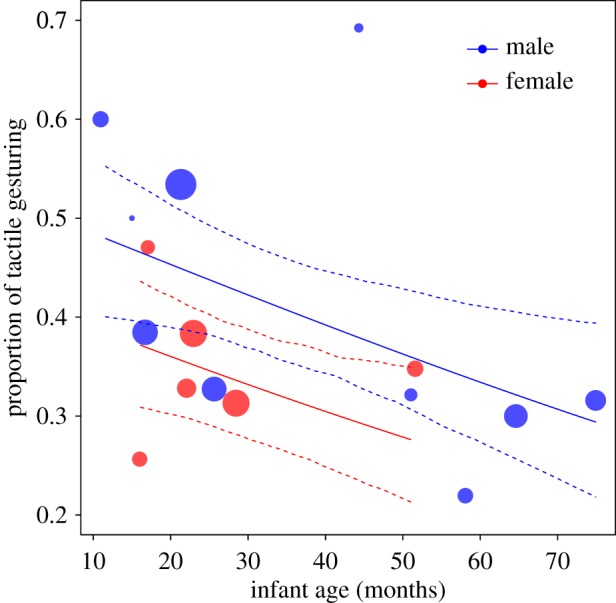
Proportion of tactile gestures employed to solicit play as a function of sex and age. Depicted are proportions, separately for each infant against its mean age. The area of the dots corresponds to the sample size per individual (range = 4–176); the solid and dashed lines represent the fitted model and confidence intervals based on all other covariates and factors centred to a mean of zero.

The two interactions between kin relationship and (between-/within-) age were non-significant in the other two models and were removed before testing the individual effects. With respect to tactile gestures, we found a clear sex difference: males produced significantly more tactile gestures to initiate play than females (sex/male: 0.387 ± 0.189, *χ*^2^ = 4.216, d.f. = 1, *p* = 0.04; figure 1). In addition, younger individuals were more likely to use tactile gestures than older ones (between-age: −0.237 ± 0.088, *χ*^2^ = 6.732, d.f. = 1, *p* = 0.009; figure 1), and tactile gestures were more likely to be produced towards mothers than other individuals (relation/mother: 0.492 ± 0.249, *χ*^2^ = 3.701, d.f. = 1, *p* = 0.054). Again, we found no effect of maternal kinship (relation/kin: −0.311 ± 0.224, *χ*^2^ = 1.559, d.f. = 1, *p* = 0.212) or site affiliation on tactile gesturing (site/*Taï South*: −0.058 ± 0.181, *χ*^2^ = 0.107, d.f. = 1, *p* = 0.744).

**Figure 2.**
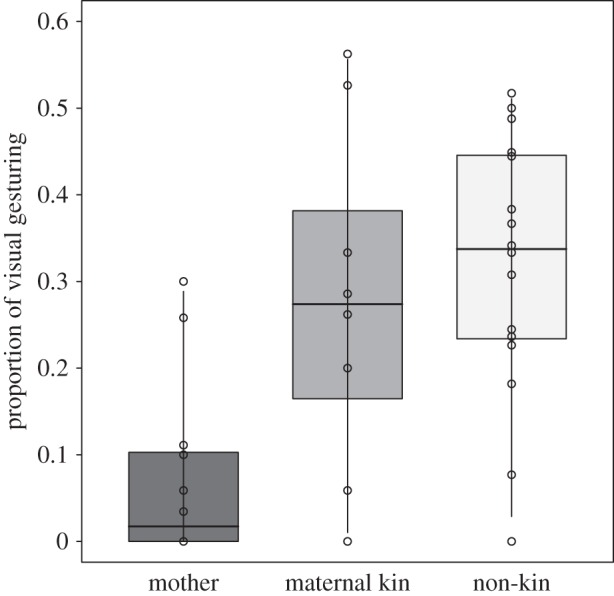
Proportion of visual gestures employed to solicit play as a function of the addressed play partner (mother, maternal kin or other non-kin conspecific). Dots represent mean proportions per infant. Indicated are median (horizontal lines), quartiles (boxes) and percentiles (2.5 and 97.5%, vertical lines). Mother: *N* = 12, maternal kin: *N* = 8 and non-kin: *N* = 16.

Visual gestures, on the contrary, were significantly more produced by older individuals (between-age: 0.241 ± 0.115, *χ*^2^ = 4.294, d.f. = 1, *p* = 0.038), and were significantly less often produced towards mothers than towards other individuals (relation/mother: −2.008 ± 0.559, *χ*^2^ = 14.123, d.f. = 1, *p* < 0.001; figure 2). Sex, maternal kinship or site affiliation had no significant effect on the production of visual gestures (sex/male: −0.369 ± 0.251, *χ*^2^ = 2.103, d.f. = 1, *p* = 0.147; relation/kin: −0.118 ± 0.335, *χ*^2^ = 0.122, d.f. = 1, *p* = 0.727; site/*Taï South*: −0.270 ± 0.226, *χ*^2^ = 1.420, d.f. = 1, *p* = 0.233). For further details on model output, see table 1.

**Table 1.** Factors influencing (*a*) audible, (*b*) tactile and (*c*) visual gesture production to solicit play. Generalized linear mixed models (GLMMs) were used with sex, age, maternal kinship and site as fixed effects, while identities of signallers, recipients and play dyads were included as random effects. Significant effects are highlighted in italics (number of observations: *N* = 1157 across 16 subjects). ^(1)^Not shown as lacking a meaningful interpretation.

	estimate	s.e.	*χ*^2^	*p-*value
(*a*) audible gesturing				
intercept	−3.083	0.552	^(1)^	^(1)^
sex (male)	−0.010	0.613	0.002	0.990
within-age	0.653	0.379	^(1)^	^(1)^
* between-age*	*0*.*748*	*0*.*209*	*11*.*256*	*0*.*001*
relation (mother)	−4.480	2.677	^(1)^	^(1)^
relation (kin)	−0.900	0.529	2.738	0.098
site (Taï South)	−0.424	0.508	0.716	0.398
* relation (mother) × within-age*	*6*.*132*	*4*.*352*	*4*.*117*	*0*.*042*
(*b*) tactile gesturing				
intercept	−0.865	0.176	^(1)^	^(1)^
* sex (male)*	*0*.*387*	*0*.*189*	*4*.*216*	*0*.*040*
within-age	0.129	0.096	1.749	0.186
* between-age*	*−0*.*237*	*0*.*088*	*6*.*732*	*0*.*009*
* relation (mother)*	*0*.*492*	*0*.*249*	*3*.*701*	*0*.*054*
relation (kin)	0.311	0.224	1.559	0.212
site (Taï South)	−0.058	0.181	0.107	0.744
(*c*) visual gesturing				
intercept	−0.550	0.220	^(1)^	^(1)^
sex (male)	−0.369	0.251	2.103	0.147
within-age	0.009	0.095	0.010	0.920
* between-age*	*0*.*241*	*0*.*115*	*4*.*294*	*0*.*038*
* relation (mother)*	*−2*.*008*	*0*.*559*	*14*.*123*	*<0*.*001*
relation (kin)	−0.118	0.335	0.122	0.727
site (Taï South)	−0.270	0.226	1.420	0.233

### 3.5. Object-associated gestures in play solicitations

**Figure 3.**
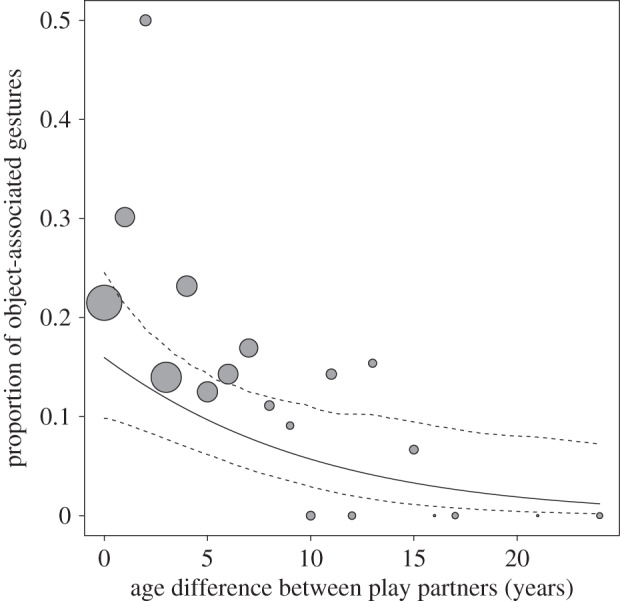
Proportion of object-associated gestures used to solicit play in relation to the absolute age difference between play partners. Depicted are proportions, separately per year of age difference. The area of the dots corresponds to the sample size for object-associated gestures per year of age difference (range = 1–242); the solid and dashed lines represent the fitted model and confidence intervals based on all other covariates and factors centred to a mean of zero.

To test whether signallers' age (within-/between-age) and sex, recipients' sex, age difference and maternal kinship affected the production of gestures accompanied by objects outside the mother–infant bond, we again used GLMMs with a binomial error function. While controlling for site, we found that the test predictors had a clear impact in our model (LRT: *χ*^2^ = 21.651, d.f. = 6, *p* = 0.001). Results showed that object-associated gestures were more frequently used with increasing age of the same infant (within-age: 0.530 ± 0.143, *χ*^2^ = 8.002, d.f. = 1, *p* = 0.005) and used more by older compared with younger infants (between-age: 0.572 ± 0.230, *χ*^2^ = 5.396, d.f. = 1, *p* = 0.020). In addition, the production of object-accompanied gestures was more likely for smaller absolute age differences between the partners (−0.090 ± 0.042, *χ*^2^ = 4.925, *p* = 0.026; figure 3). No other effects in the model (signaller's sex, recipient's sex, maternal kinship or site) reached significance (table 2*a*).

**Table 2.** Factors influencing (*a*) object-associated gesturing and (*b*) self-handicapping to solicit play. Generalized linear mixed models (GLMMs) were used with signaller's sex and age, recipient's sex, age difference relative to play partner, maternal kinship and site as fixed effects, while identities of signallers, recipients and play dyads were included as random effects. Significant effects are highlighted in italics (number of observations: (*a*) *N* = 959 across 16 subjects; (*b*) *N* = 1264 across 32 subjects). ^(1)^Not shown as lacking a meaningful interpretation.

	estimate	s.e.	*χ*^2^	*p-*value
(*a*) object-associated				
intercept	−1.223	0.482	^(1)^	^(1)^
sex (male)	−0.450	0.478	0.833	0.361
* within-age*	*0.530*	*0.143*	*8.002*	*0.005*
* between-age*	*0.572*	*0.230*	*5.396*	*0.020*
sex of recipient (male)	−0.520	0.327	2.616	0.106
* age difference (abs.)*	*−0.090*	*0.042*	*4.925*	*0.026*
relation (kin)	−0.418	0.425	0.953	0.329
site (Taï South)	0.485	0.455	1.118	0.290
(*b*) self-handicapping				
intercept	−8.470	1.320	^(1)^	^(1)^
sex (male)	1.080	0.692	1.478	0.224
within-age	−0.155	0.254	0.360	0.548
* between-age*	*−0.477*	*0.230*	*3.933*	*0.047*
sex of recipient (male)	−0.103	1.011	0.010	0.919
relation (kin)	−0.206	0.172	1.115	0.291
age difference (−)	−5.273	4.562	2.093	0.148
* age difference (+)*	*4.751*	*1.065*	*9.341*	*0.002*
* site (Taï South)*	*2.041*	*0.849*	*5.399*	*0.020*

### 3.6. Self-handicapping in play solicitations

**Figure 4.**
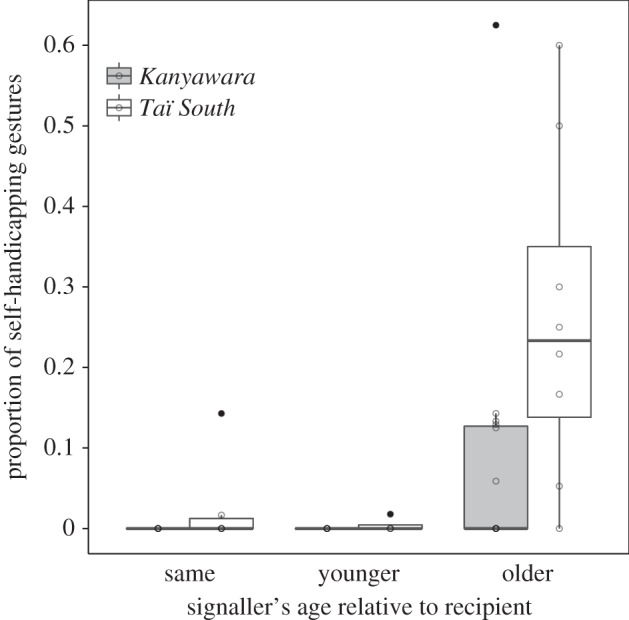
Proportion of self-handicapping gestures produced towards same-aged, younger and older play partners at *Kanyawara* (*N* = 27) and *Taï South* (*N* = 47) study sites, respectively. Indicated are median (horizontal lines), quartiles (boxes), percentiles (2.5% and 97.5%, vertical lines) and outliers (dots). Total number of individuals included: 33.

Finally, we used a sixth GLMM with a binomial error structure to investigate the influence of signallers' age (within-/between-age) and sex, recipients' sex, age difference (older versus younger partners) and maternal kinship on the production of self-handicapping gestures, while controlling for study site. The test predictors had a significant impact on the occurrence of self-handicapping gestures (LRT: *χ*^2^ = 18.369, d.f. = 7, *p* = 0.010). Chimpanzees used self-handicapping signals significantly more often towards recipients that were younger in age (age difference (+): 4.751 ± 1.065, *χ*^2^ = 9.341, d.f. = 1, *p* = 0.002; figure 4). Additionally, they were more likely to be produced in younger individuals (between-age: −0.477 ± 0.230, *χ*^2^ = 3.933, d.f. = 1, *p* *=* 0.047). *Taï South* chimpanzees produced more self-handicapping gestures than *Kanyawara* chimpanzees (site/*Taï South*: 2.041 ± 0.849, *χ*^2^ = 5.400, d.f. = 1, *p* = 0.020). None of the other effects in the model (signaller's sex, recipient's sex and maternal kinship) reached significance (table 2*b*)

## 4. Discussion

This is the first study that systematically investigated gestures employed to solicit play in chimpanzees of two different subspecies and communities in two chimpanzee communities (*Kanyawara*, Kibale National Park, Uganda, and *Taï South*, Taï National Park, Côte d'Ivoire) living in their natural environments. Our aim was to examine the influence of demographic factors and kinship on gestural play solicitations via ‘pure’, object-associated and self-handicapping gestures. To target these aims, we analysed play interactions to address the following three questions: first, do age and sex of the play partners influence the production of audible, tactile and visual gestures? Second, to which extent does the kin relationship with the play partner influence the modality of gestures used to initiate play? Third, do chimpanzees take into consideration recipients' attributes when using object-associated and self-handicapping gestures? Overall, our results showed that both signallers' age and sex as well as a prevailing kin relationship with the play partner significantly influenced the category of being employed. Moreover, we found that object-associated and self-handicapping gestures had a crucial importance for initiating play with same-aged and younger play partners, respectively. These findings imply that chimpanzees are able to adjust their use of gesture flexibly to distinct attributes of conspecifics. We thus expand recent findings on the degree of gestural flexibility and underlying cognitive tool-kits in chimpanzees and great apes by showing that chimpanzees not only take into consideration attentional states of recipients [21,27,28] and social contexts [21] but also distinct characteristics of their conspecifics. In the following paragraphs, we will discuss each of our research questions and the related findings in detail.

In a first step, we compiled repertoires of play-solicitation gestures in relation to age class and study site resulting in around 50 gesture types used by chimpanzees. This result is in line with previous studies showing that the play context comprises a particularly large number of gesture types [21]. We also strengthen observations on the behaviour of free-ranging chimpanzees, showing that play-soliciting gestures in chimpanzees consist of a remarkable variety of behavioural patterns [4]. This included audio–visual attention-getters [78], self-handicapping signals [55] and elements of both solitary and social play [4]. Intriguingly, many of the reported attention-getting gestures (e.g. poke, slap ground and hit with object) do not involve specific information about the context of play, which might be rather expressed through the context-typical ‘play face’ and/or the play postures. Tomasello *et al.* [78] highlighted this signal combination as vital element in chimpanzee communication. Since attention-getting gestures might be linked to an understanding of the recipient's intention and attention, Tomasello [79] argued that this gesture class is particularly novel and complex in the animal kingdom (however, note that great apes' abilities to understand others' intentions is still subject to an ongoing debate; [80,81]). Moreover, our results showed that age classes varied considerably in signal repertoires, with decreasing repertoire sizes used by individuals of older age classes. This is corroborated by earlier studies showing that gestural repertoires in several ape species increase sharply as infants develop, and then decrease again in adult individuals [26,28,29]. Since these studies demonstrated that many gestures are used in the play context, this highlights the major role of play for immature individuals as opposed to adults [82]. It is noteworthy that gesture counts, regardless of category, were always (except for self-handicapping gestures, see below) slightly larger in individuals of the *Kanyawara* community despite comparable observation times across sites. However, this difference should not be overstated since the amount of recorded play interactions was considerably larger for *Kanyawara* compared with *Taï South* (see Material and methods). We controlled for site effects in all our analyses and could thus show that study site had no significant influence on our results. Self-handicapping gestures comprised the only exception, because they were significantly more often produced by members of the *Taï South* community. We found evidence for five group-specific gesture types, yet each of these has been observed in (at least) one other community of Eastern chimpanzees [58,60] and can, therefore, not be considered as novel gestures unique to the respective community. However, since these communities do not exchange members, they could provide evidence for parallel invention. For instance a distinct grooming solicitation gesture used by chimpanzees, the directed scratch, has so far only been reported from the *Ngogo* and *Budongo* communities in Uganda [61,83]. It is thus likely that the underlying developmental process (social negotiation, see below) from a non-communicative, undirected self-scratch into a communicative behaviour is the same [84]. The only other gesture type with geographical variation in wild chimpanzees, the grooming hand-clasp, has been suggested to demonstrate cultural transmission within a group [85,86]. It is, however, not clear whether the hand-clasp qualifies as gesture, since its communicative function is still debated [82] and it might simply act as a stabilizing tool. Our findings provide further support for the *social negotiation hypothesis* proposed by Fröhlich *et al.* [36] which postulates that gestures originate from the exchange of social behaviours resulting in a mutual recognition that (i) certain behaviours can be used communicatively, (ii) carry distinct meaning linked to particular social contexts, and (iii) are produced to achieve distinct goals. However, further fine-grained analyses of great ape gestural production are necessary to reveal the form of gestures in relation to age, context and social partner. In addition, we cannot completely rule out that the group-specific gesture types found resulted from our study design focusing only on interactions of infant chimpanzees with conspecifics.

With regard to the first question addressing the effect of age and sex on the production of distinct gesture categories, we found that visual and audible gestures were employed more frequently with increasing infant age. The production of tactile gestures was however higher in younger individuals. These findings are in line with results of a recent study at the same sites [36], at *Gombe*, Tanzania [41,87], and another study conducted in captivity [40]: chimpanzee infants become intentional agents throughout development, capable of manipulating the attentional and maybe also the mental states of their conspecifics [28,41,61]. To investigate the influence of sex differences, we firstly analysed whether males and females differ concerning the intensity of play that they are initiating. This enabled us to rule out that sex differences in signalling merely result from differences in play intensity for males and females. We found that the two sexes did not differ significantly with regards to the intensities of the initiated play bouts. In terms of gestural solicitations, however, males used significantly more tactile gestures than females. Tactile gestures differ from visual gestures in both physical effectiveness and potential demonstration of physical strength. We thus interpret these results as evidence for a sex difference in signal directness in terms of the level of physical contact involved. In addition, a recent study by Lonsdorf *et al.* [15] showed pronounced sex differences in social play interactions of chimpanzees, with males showing higher playing rates at earlier ages, a more diverse set of social partners and a larger frequency of interaction with adult males. They thus argued for the higher importance of socialization for young males given the importance of social bonds and apprenticeship during development and social dominance in adulthood [88]. In addition, this learning period might have crucial implications for the reproductive success in a given community: it could result in moving around with one of the highest ranking males and being his ‘apprentice’, having access to valuable food resources, resulting in stable and linear growth and higher reproductive success in the longer term [89,90]. In light of the social matrices of chimpanzees, it can be reasonably assumed that investment in play during development can take priority over physical development for skill acquisition [2]. A similar phenomenon has been reported for human infants concerning general play behaviour, with male infants exhibiting more independent exploratory and vigorous play behaviour than female infants [91,92]. However, virtually nothing is known about whether humans and primates differ between sexes in terms of their usage of communicative signals to *initiate* or *solicit* social play. Studies have shown that play signalling in humans is reduced to the face, taking the form of laughter and smiling [93]. Nonetheless, more research is needed to elucidate the role of body postures and non-manual gestures in this context. So far, most human gesture research has focused on visual signals in space [62,94,95], which might be one crucial factor responsible for the diverging gesture definitions in human and primate gesture research. Nevertheless, our results indicate that biological factors, that is, the selection pressures based on the sex-specific behavioural roles [96], rather than environmental factors (i.e. socialization processes), may influence sex differences in human play behaviour [14].

Regarding the influence of relationship (mother, maternal kin or non-kin) to the play partner, we found that tactile gestures were more likely to be used in interactions with mothers than with other individuals (i.e. maternal kin and non-kin partners). Visual gestures on the other hand, which were more commonly used with increasing infant age, were produced more frequently to solicit play with conspecifics than with mothers. We ruled out that this increase is just an effect of infants gesturing more frequently towards conspecifics with increasing age, by showing that there is no interaction between kin relationship and age. Thus, we demonstrated that both results, visual gestures being more employed with increasing age on the one hand and towards conspecifics on the other, are independent from each other. The same independence was confirmed concerning the use of tactile gesturing. For audible gesturing, however, we found a more frequent usage towards mothers with increasing age of an individual (within-age), while the effect of between-age (older versus younger infants) was independent from the one of kin relationship.

Hence, our results are partly in line with our predictions regarding higher frequencies of tactile signals within mother–infant interactions, while a maternal kin relationship between the play partners, as in the case of siblings, did not have an influence. Considering the difference between signalling within and outside the mother–infant bond, it appears that the mother–infant attachment is a stronger predictor than familiarity based on established predictable outcomes. Nonetheless, some unrelated individuals could be very ‘familiar’ to each other due to the frequent association of their mothers, which may significantly impact upon their communicative exchanges. However, because we did not collect data on affiliative relationships, we cannot test for this potential effect on play solicitation. Plooij [41] described a developmental shift from physical acts without social-communicatory intention to intentional tactile acts in interactions between chimpanzee infants and their mothers at *Gombe*, Tanzania. During this shift, the infant learns to use tactile behaviours, whose meanings have been established in earlier sessions, not only to *maintain* an interaction but also to *initiate* it. Our findings support the notion that the communicative development of great apes is supported by the infant leaving the ‘security range’ provided by the mother and entering its complex social environment [36,41]. Play interactions with peers and other ‘non-mother’ individuals may serve as essential grounds for experimentation, where great apes can test for the effectiveness of and practise intentional gestures that might gain vital importance in their adult life [87].

Intriguingly, about one-fifth of all gestures used to initiate or reinitiate play were accompanied by mobile or immobile objects. This behaviour was more common with advancing development and increasing infant age, probably due to growing proficiency in object handling. A recent study of chimpanzees and bonobos in the wild showed that chimpanzee infants start to manipulate objects as early as from nine months of age [97]. These object manipulations became more diverse and ‘tool-like’ (i.e. sticks) with increasing infant age, which was interpreted as preparation for tool use in adulthood [97]. However, one striking finding of our study is that this subset of gestures seems to play a larger role in interactions with same-aged peers, and a lesser role in interactions with considerably younger or older individuals (i.e. play partners with larger absolute age difference). This result strongly supports the view of play as training ground for social interactions in adult life. Young chimpanzees are possibly much more confident to use additional ‘tools’ of their communicative tool-kit if their play partners are of similar size and developmental stage. These interactions provide a safe environment and playground to train them for later, possibly more dangerous situations. Since the *Taï South* communities are renowned for their sophisticated tool-using technique of nut-cracking [13], it is surprising that gesture-object combinations do not play a larger role in *Taï South* chimpanzees. In a comparison between the long-term study sites of *Taï South*, *Mahale* and *Gombe*, Boesch & Boesch [98] found that sticks are commonly used and prepared at all three sites, while *Taï South* chimpanzees modified the material more before using it. Moreover, only *Taï South* chimpanzees pounded objects with tools and combined two different tool uses to get access to one food item [98]. However, the tool-using profiles of the two communities are distinctively different, and to our knowledge there is thus far no evidence that *Kanyawara* chimpanzees differ substantially from those in *Taï South* in terms of frequency of overall tool usage [99]. Nevertheless, the results obtained in this study might corroborate to some extent the complementation theory of language and tool use discussed in relation to human evolution [100].

Turning to self-handicapping gestures, we found that these were predominantly used to solicit play with younger, as opposed to older or same-aged, social partners. In addition, this behaviour was more common in young chimpanzees, indicating that this communicative strategy plays a larger role for juveniles and/or young sub-adults. These results thus expand findings by Flack *et al.* [23] on a captive group of chimpanzees, which showed that with larger age difference between play partners, the older play partner was more likely to play at lower intensity. They also reported a significant relation between the frequency with which an older play partner emitted play signals (e.g. play face, laughing) and the proximity of the younger play partner's mother, especially when play intensity was higher. If a young chimpanzee observes an older individual engaging in a compromising posture or performing a self-handicapping movement, it may understand that this potential playmate poses no threat. That is, an individual lying in a supine position cannot attack promptly, and an individual shaking its head cannot leap precisely, because ‘its judgement of distance and direction is momentarily blurred’ [19, p. 146]. It has been suggested that self-handicapping strategies crucially limit the risk of harming younger play partners, serve to maintain the playful mood and prevent mother's intervention [35]. These observations led Flack *et al.* [23] to conclude that chimpanzees are able to perceive customary social rules about play. The causes underlying the inter-site difference in self-handicapping signals, which were more frequently produced in *Taï South* chimpanzees, remains elusive. One explanation may be that, due to the smaller community size of *Taï South*, the same individuals might interact more frequently and form better-established play dyads, resulting in a more common usage of self-handicapping strategies.

The means–end dissociation between signal and context is a key hallmark of flexible signal use [29]. Our study thus demonstrated signal flexibility in chimpanzees with regard to multiple gesture types used for a single communicative function (play solicitation). Since many of the play-soliciting gesture types described in our study have been shown to be employed in entirely different social contexts in previous studies [29,60,101], we could also show that single gesture types can be used for different communicative purposes. Hobaiter & Byrne [83] recently found that play-related meanings of gestures occurred with high generality, that is they were not dependably communicated by the outcome in other, ‘serious' contexts. Flexible communication, as demonstrated by gestural play solicitations of chimpanzees in this study on an individual and a group level, is deficient of a one-to-one correspondence between single signals and responses, and assumed to involve higher cognitive complexity than the reflexive signals tightly linked to single contexts commonly found in the animal kingdom [82,102]. Intriguingly, a number of studies on great apes showed that social play is the context in which they test and practise the majority of gestures [21]. Hobaiter & Byrne [83] thus argued that play gestures are often not related to their normal meaning. Moreover, play signals mediating the play bout not only include gestures and body postures, as investigated in our study, but also facial expressions, spatial cues and relaxedness of movement. An exciting avenue for future studies thus would be to examine how different types of signals in play and other social contexts are integrated to achieve communicative goals and resolve ambiguity of signal meanings. Importantly, the overwhelming body of work on great ape gestures still stems from captive environments [103], which necessarily suffered from human intervention with the natural communication system [104]. However, we can only understand the communicative complexity of our closest living relatives if we also consider between-site variability [104]. Additional studies including more communities, thereby taking into account potential within-species variability, are needed to gain in-depth understanding of the cognitive mechanisms underlying ape communication.

## 5. Conclusion

In sum, by investigating the impact of demographic factors and kin relationships on play interactions in two different chimpanzee communities, our study has provided hitherto undocumented findings on how age, sex and a prevailing kin relationship to the recipient can influence gestural usage in natural environments. We showed that chimpanzee infants take into account distinct attributes of the play partner, using a higher frequency of visual and audible gestures towards individuals other than their mothers and a lower frequency of tactile (risky) gestures. Our study demonstrated that both object-associated and self-handicapping gestures are essential parts of the communicative tool-kit of chimpanzees that serve different functions depending on the age difference to the respective play partner. These findings further highlight the role of the play context as training ground for tool use and maintaining social relationships in later life. Moreover, this study provides evidence for a mutual construction of gestures by interactants and the cognitive ability to flexibly adjust these communicative means to social circumstances and individual matrices. We study thus demonstrated gestural flexibility in great apes at an unprecedented scale. Although we will never be able to entirely grasp chimpanzee behavioural plasticity in its full complexity, systematic comparisons of the behaviour between communities and between subspecies are a vital step into this intriguing endeavour.

## Supplementary Material

ESM_1: Supplementary tables and figures

## Supplementary Material

ESM_2: Data file for models 1-5

## Supplementary Material

ESM_3: Date file for model 6

## Footnotes

1 From here gestures are depicted in small capitals.

## Data accessibility

The datasets supporting this article have been uploaded as part of the electronic supplementary material.
