# Supplementary material for: Play-solicitation gestures in chimpanzees in the wild: flexible adjustment to social circumstances and individual matrices
Source: R Soc Open Sci. 2016 Aug 24;3(8):160278. doi: 10.1098/rsos.160278 (PMC5108953; doi:10.1098/rsos.160278)
Supplement: ESM_1: Supplementary tables and figures [file rsos160278supp1.docx]

**Play-solicitation gestures in chimpanzees in the wild: Flexible adjustment to social circumstances and individual matrices**

Marlen Fröhlich^1, *^, Roman M. Wittig^2,3^ & Simone Pika^1^

^1^Max Planck Institute for Ornithology, Humboldt Research Group ‘Evolution of Communication’, Eberhard-Gwinner-Strasse 9, D-82319 Seewiesen, Germany

^2^Max Planck Institute for Evolutionary Anthropology, Primatology Department, Deutscher Platz 6, D-04103 Leipzig, Germany

^3^Taï Chimpanzee Project, Centre Suisse de Recherches Scientifiques, BP 1303, Abidjan 01, Côte d’Ivoire

**Supplementary Material**

Table S1 Information on observed mother-infant dyads with respective observation time and raw data set. The last line provides the total sample size for each column (P1/P2 first/second period of data collection).

|  | **Infant (ID)** | **Sex** | **Age P1 [months]** | **Age P2 [months]** | **Video-recorded play interactions [hours]** |  |
| --- | --- | --- | --- | --- | --- | --- |
| **Kanyawara** | Winza (WZ) | M | 9–11 | 21–23 | 11.0 |  |
|  | Tembo (OB) | M | 13–15 | 25–27 | 13.9 |  |
|  | Mango (MM) | F | 13–15 | 25–27 | 4.9 |  |
|  | Lily (LL) | F | 3–5^a^ | 15–17 | 5.1 |  |
|  | Thatcher (TR) | F | 16–18 | 28–30 | 13.4 |  |
|  | Basuta (BT) | M | 32–34 | 44–46 | 1.2 |  |
|  | Gola (OL) | F | 48–50 | 60–62 | 6.9 |  |
|  | Moon (MN) | M | 50–52 | 62–64 | 2.7 |  |
|  | Quiver (QV) | M | 55–57 | 67–69 | 1.7 |  |
|  | Wallace (WC) | M | 55–57 | 67–69 | 5.6 |  |
| **Taï South** | Mohan (MH) | F | 10–12 | 22–24 | 12.6 |  |
|  | Iniesta (IN) | M | N/A | 10­–12 | 9.3 |  |
|  | Solibra (SL) | M | 15–17 | 27–29 | 14.6 |  |
|  | Jeff (JF) | M | 15^b^ | N/A | 0.4 |  |
|  | Kayo (KY) | F | 19–21 | 31–33 | 14.9 |  |
|  | Ithaka (IT) | M | 64–66 | 72–74 | 12.3 |  |
| N | 16 | 6:10 | 14 | 15 | 8.2 ± 5.2 |  |

^a^ P1 not included since infant was too young

^b^ deceased on Nov 1, 2012

Table S2 Gesture types produced to initiate play in chimpanzees identified in this and other studies on wild groups in Sonso^[1](#_ENREF_1)^; Gombe[^2^](#_ENREF_2) and Mahale[^3^](#_ENREF_3).

| **Gesture type** | **Definition (this study)** | **Sonso** | **Gombe** | **Mahale** |
| --- | --- | --- | --- | --- |
| ***Audible*** |  |  |  |  |
| shake branch | Signaller moves attached plant parts repeatedly back and forth | Object move | Branch shaking | Shake branch |
| slap ground | Signaller hits ground with flat palm of his hand | Slap object | Slap ground | Slap branch |
| stomp | Signaller contacts the surface stood upon with short, audible movement of his foot  short hard audible  being stood upon | Stomp | Stamping | Stamp |
| ***Tactile*** |  |  |  |  |
|  |  |  |  |  |
| bite | Signaller holds party of recipient’s body between the teeth | Bite | Mouthing | Mouth |
| bite pretend | Signaller contacts recipient’s body with open mouth (teeth not closed) | - | Open mouth kiss | Open mouth kiss |
| drum on | Signaller contacts recipient with short hard, solid contact of alternate palms against body | Drum other | - | - |
| embrace | Signaller puts both arms around the recipient’s body | Embrace | Embracing | Embrace full |
| finger in mouth | Signaller puts fingers repeatedly over the mouth region of recipeint | Moth stroke | - | - |
| grab | Signaller closes fingers firmly around a body part of the recipient | Grab | Grab | Grab |
| grab foot | Signaller closes fingers firmly around a foot of the recipient |  |  |  |
| grab-push | Signaller is maintains firm hand contact to bring the recipient to move  current position | Grab-pull | - | Pull |
| head butt | Signaller pushes the recipient’s body solidly and briefly with top of the head | Head butt | - | - |
| hit | Signaller contacts the recipient’s body solidly and briefly with palm of hand | Slap other | Slapping, hit | Slap |
| hit with obj | Signaller contacts the recipient’s body solidly and briefly with hand-held object | Hit with object | - | Club |
| hold onto | Signaller grasps and maintains physical contact with recipient | Grab and hold | - | Grasp hand |
| kick | Signaller contacts the recipient’s body solidly and briefly with foot | Kick | Kick | Kick |
| poke | Signaller pushes of one of more fingers solidly and shortly onto recipient’s body | Poke | Poking | Poke |
| pull | Signaller moves recipient’s body part towards himself | Pull | Pull | Pull |
| pull object | Signaller moves object the recipient is holding onto (e.g., branch, stick) towards himself |  |  |  |
| push | Signaller uses limbs or body to bring recipient in direction of movement. | Directed push | Pull towards | Push ahead |
| rub genitals | Signaller pushes/rubs own genitals against the recipient’s body | Rump rub (body) | Rub | Rub |
| steal object | Signaller removes quickly an object (“toy”) from recipient while in his visual field | - | - | - |
| step on | Signaller steps on recipient | - | - | Step on |
| tickle | Signaller makes tickling movements with the fingers on the recipient’s body | - | Tickle | Tickle |
| touch, long | Signaller makes long (> 2 seconds) contact with recipient using palm and/or fingers | Hand on | Touch | Touch |
| touch, short | Signaller makes short (< 2 seconds) contact with recipient using palm and/or fingers. | Touch other | Touch | Touch |
| wrap arms | Signaller wraps arms around head/neck of recipient while turned around | - | Hands around head | - |
| **Table S2** continued | |  |  |  |
| **Gesture type** | **Definition (this study)** | **Sonso** | **Gombe** | **Mahale** |
| ***Visual*** |  |  |  |  |
| bob | Signaller moves head and upper body rapidly up and down | Head nod | Head tipping | Bob |
| flail arm | Signaller moves arm swiftly towards the recipient | Arm wave | - | Flail arm |
| hide self | Signaller covers own face with hands and/or arms | Hide face | - | - |
| hang sloth | Hanging underneath a branch in front or above the recipient | - | - | Hang in sloth position |
| lie down | Signaller lies on back, exposing ventral body | Roll over | Lie down on back | - |
| lie spread legs | Signaller lies in supine position and moves quickly arms and/or legs | Roll over | - | Lie supine and shake arms and legs |
| look | Signaller gazes at recipient (> 2 seconds) | Look | Wait | Look; Wait |
| look back | Signaller stops with body orientated in direction of movement and looks back (or down) at recipient | - | - | Look back |
| look through thighs | Signalling stands quadrupedally, lowers head and looks at recipient though his spread legs | - | - | Look between  thighs |
| present back | Signaller turns back towards recipient | Present climb on | Lowering back | - |
| present leg | Signaller presents leg or foot to the recipient | Foot present | - | - |
| raise arm | Signaller raises arm vertically above head | Arm raise | Arm raise | Raise arm |
| raise forearm | Signaller raises forearm vertically in the air | Arm raise | Arm raise | Raise arm |
| reach | Signaller extends arm towards recipient | Reach | Extend hand | Extend hand |
| shake head | Signaller moves head rapidly from side to side | Head shake | - | Shake head |
| somersault | Signaller rolls forward or backward in a complete revolution with the knees bent and  the feet coming over the head | Somersault | Somersault | Somersault |
| staksy walk | Signaller walks in small strained steps with a rounded back and its head slightly bent down | Walk (stiff) | Play-walk | Play walk |
| stand up | Signaller briefly stands upright in bipedal posture | - | - | Stand bipedal |
| swing | Signaller hangs from vine or branch by arms and swing back and forth or up and down | - | - | Swing |
| swing obj | Signaller shakes unattached object (e.g. plant material, stick, stone) in hand | Arm swing with object | - | Shake detached branch |
| tilt head | Signaller inclines head to one side while standing quadruped | - | - | Tilt head |

Table S3 Gesture and action types used by different age classes and communities. Gesture types in bold represent group-specific types.

| **Gesture category** | **Community** | **Kanyawara** | | | | **Taï South** | | | | **TOTAL** |
| --- | --- | --- | --- | --- | --- | --- | --- | --- | --- | --- |
|  | **Age Class** | **INF** | **JUV/SUB** | **ADT** | **All** | **INF** | **JUV/ SUB** | **ADT** | **All** | **All** |
|  | **N Individuals** | **10** | **10** | **11** | **31** | **6** | **8** | **4** | **18** | **49** |
| Gesture, audible | shake branch | 3 | 2 | 1 | **6** | 3 | 4 | 0 | **7** | **13** |
|  | slap ground | 7 | 2 | 0 | **9** | 2 | 1 | 0 | **3** | **12** |
|  | stomp | 3 | 1 | 0 | **4** | 1 | 2 | 0 | **3** | **7** |
| Gesture, tactile | bite | 7 | 1 | 5 | **13** | 4 | 2 | 2 | **8** | **21** |
|  | bite pretend | 4 | 1 | 6 | **11** | 3 | 0 | 2 | **5** | **16** |
|  | drum on | 3 | 0 | 0 | **3** | 3 | 0 | 0 | **3** | **6** |
|  | embrace | 3 | 1 | 0 | **4** | 2 | 0 | 1 | **3** | **7** |
|  | finger in mouth | 4 | 0 | 1 | **5** | 0 | 0 | 1 | **1** | **6** |
|  | grab | 6 | 2 | 4 | **12** | 4 | 2 | 2 | **8** | **20** |
|  | grab foot | 6 | 3 | 5 | **14** | 4 | 1 | 3 | **8** | **22** |
|  | grab-push | 2 | 0 | 1 | **3** | 3 | 0 | 1 | **4** | **7** |
|  | **head butt** | 6 | 1 | 0 | **7** | 0 | 0 | 0 | **0** | **7** |
|  | hit | 8 | 2 | 1 | **11** | 5 | 2 | 0 | **7** | **18** |
|  | hit with obj | 4 | 0 | 0 | **4** | 1 | 0 | 0 | **1** | **5** |
|  | hold onto | 6 | 0 | 0 | **6** | 3 | 1 | 0 | **4** | **10** |
|  | kick | 5 | 2 | 3 | **10** | 0 | 1 | 1 | **2** | **12** |
|  | poke | 7 | 7 | 8 | **22** | 3 | 1 | 3 | **7** | **29** |
|  | pull | 9 | 3 | 3 | **15** | 5 | 3 | 2 | **10** | **25** |
|  | pull object | 6 | 3 | 4 | **13** | 3 | 3 | 2 | **8** | **21** |
|  | push | 5 | 2 | 4 | **11** | 3 | 0 | 1 | **4** | **15** |
|  | rub genitals on | 2 | 0 | 0 | **2** | 1 | 0 | 0 | **1** | **3** |
|  | steal object | 3 | 0 | 1 | **4** | 2 | 1 | 0 | **3** | **7** |
|  | step on | 7 | 0 | 0 | **7** | 3 | 0 | 0 | **3** | **10** |
|  | tickle | 4 | 3 | 8 | **15** | 0 | 1 | 2 | **3** | **18** |
|  | touch, long | 5 | 0 | 2 | **7** | 1 | 0 | 0 | **1** | **8** |
|  | touch, short | 9 | 5 | 7 | **21** | 5 | 2 | 3 | **10** | **31** |
|  | wrap arms | 3 | 0 | 0 | **3** | 2 | 0 | 0 | **2** | **5** |
| Gesture, visual | **hide self** | 2 | 1 | 0 | **3** | 0 | 0 | 0 | **0** | **3** |
|  | bob | 2 | 0 | 0 | **2** | 1 | 0 | 0 | **1** | **3** |
|  | flail arm | 8 | 3 | 1 | **12** | 3 | 3 | 0 | **6** | **18** |
|  | hang sloth | 3 | 1 | 0 | **4** | 1 | 2 | 0 | **3** | **7** |
|  | lie down | 1 | 2 | 0 | **3** | 0 | 4 | 0 | **4** | **7** |
|  | lie w spread legs | 4 | 4 | 1 | **9** | 3 | 5 | 1 | **9** | **18** |
|  | look | 6 | 1 | 0 | **7** | 3 | 4 | 0 | **7** | **14** |
|  | look back at | 6 | 0 | 0 | **6** | 3 | 0 | 0 | **3** | **9** |
|  | look through thighs | 2 | 2 | 1 | **5** | 0 | 3 | 0 | **3** | **8** |
|  | **present back** | 2 | 1 | 0 | **3** | 0 | 0 | 0 | **0** | **3** |
|  | **present leg** | 4 | 3 | 1 | **8** | 0 | 0 | 0 | **0** | **8** |
|  | raise arm | 7 | 1 | 0 | **8** | 2 | 0 | 0 | **2** | **10** |
|  | raise forearm | 4 | 2 | 1 | **7** | 0 | 1 | 0 | **1** | **8** |
|  | reach arm | 8 | 7 | 2 | **17** | 4 | 2 | 1 | **7** | **24** |
|  | **shake head** | 0 | 0 | 0 | **0** | 1 | 1 | 0 | **2** | **2** |
|  | somersault | 4 | 2 | 1 | **7** | 1 | 0 | 0 | **1** | **8** |
|  | staksy walk | 4 | 0 | 0 | **4** | 1 | 2 | 0 | **3** | **7** |
|  | stand up | 2 | 0 | 0 | **2** | 1 | 0 | 0 | **1** | **3** |
|  | swing | 6 | 0 | 0 | **6** | 4 | 1 | 0 | **5** | **11** |
|  | swing obj in hand | 7 | 1 | 0 | **8** | 2 | 1 | 0 | **3** | **11** |
|  | tilt head | 1 | 1 | 0 | **2** | 1 | 0 | 0 | **1** | **3** |
|  | **N Gesture types** | 47 | 32 | 24 | **47** | 39 | 27 | 16 | **44** | **48** |
|  |  |  |  |  |  |  |  |  |  |  |

Table S4 Factors influencing play intensity after play-soliciting gesture. A Generalized Linear Mixed Model (GLMM) was used with sex, age, kin relationship and site as fixed effects, while identities of signaller, recipient and play dyad were included as random effects. (Number of observations: N = 1155 across 16 subjects).

| **Play intensity** | Estimate | S.E. | *χ*^2^ | *P* |
| --- | --- | --- | --- | --- |
| Intercept | 0.498 | 0.083 | ^(1)^ | ^(1)^ |
| Sex [male] | 0.004 | 0.090 | 0.002 | 0.968 |
| Within-age | 0.023 | 0.032 | 0.472 | 0.492 |
| **Between-age** | **0.152** | **0.048** | **8.210** | **0.004** |
| **Relation [mother]** | **–0.281** | **0.089** | **9.892** | **0.002** |
| Relation [kin] | –0.063 | 0.104 | 0.378 | 0.539 |
| Site [Taï] | –0.119 | 0.090 | 1.783 | 0.182 |

Bold values indicate *p* < 0.05

^(1)^ Significance test not indicated because it has no meaningful interpretation

Figure S1 Cumulative gestural repertoire used in joint travel interactions plotted against days of observation for the two study sites *Kanyawara* (A) and *Taï South* (B).

Figure S2 Influence of sex on the intensity of play that was initiated. We differentiated between low (soft play, touching, tickling), intermediate (wrestling and biting), and high intensity (rough and tumble) play interactions (*N* females= 6, *N* males = 10).

References

1 Hobaiter, C. & Byrne, R. The gestural repertoire of the wild chimpanzee. *Animal Cognition* 14, 745-767 (2011).

2 Goodall, J. *The chimpanzees of Gombe, Patterns of Behaviour*. (The Belknap Press of Harvard University Press, 1986).

3 Nishida, T., Zamma, K., Matsusaka, T., Inaba, A. & McGrew, W. C. *Chimpanzee behavior in the wild: an audio-visual encyclopedia*. (Springer Science & Business Media, 2010).
